# Rabbi Moshe Ben Maimon, Better Known as Rambam (1138–1204): Physician, Philosopher, and Adjudicator

**DOI:** 10.7759/cureus.71591

**Published:** 2024-10-16

**Authors:** David Ezra, Joe Iwanaga, R. Shane Tubbs

**Affiliations:** 1 School of Nursing Sciences, The Academic College of Tel Aviv Jaffa, Tel Aviv Jaffo, ISR; 2 Department of Anatomy and Anthropology, Tel Aviv University, Tel Aviv, ISR; 3 Department of Physical Anthropology, Natural History Museum, Cleveland, USA; 4 Department of Neurosurgery, Tulane University School of Medicine, New Orleans, USA; 5 Department of Structural and Cellular Biology, Tulane University School of Medicine, New Orleans, USA; 6 Department of Neurosurgery, Ochsner Neuroscience Institute, Ochsner Health System, New Orleans, USA; 7 Department of Anatomical Sciences, St. George's University, St. George's, GRD

**Keywords:** adjudicator, moshe ben maimon, philosopher, physician, rambam

## Abstract

Rabbi Moshe Ben Maimon, known as Maimonides (Hebrew name is Rambam), lived from 1138 to 1204. He was one of the important philosophers, adjudicators, and physicians of the Jews. Rambam's knowledge of Jewish laws and his being a philosopher helped him in his third discipline, medicine. One of his perspectives was combining theology with philosophy, especially medicine. With that ability, he pioneered his multidisciplinary way of thinking and acting. Rambam was also a pioneer in the medical fields, especially anatomy, physiology, and clinical knowledge, and inspired many generations in the Middle Ages. Rambam was esteemed and respected not only by Jews but also by other religions and nationalities. There is a notable saying describing Rambam: "From Moses (Moses, the prophet) to Moshe (Moses, the Rambam), there were none like Moses." He was nicknamed the "Big Eagle". Rambam was called "Mossa Ibn Maimon" by the Arabs and "Maimonides" by the Europeans, derived from the Greek word "Moiasis Maimonidis". Herein, we focus solely on Rambam's contributions to medicine since his work, activities, and knowledge are very complex to summarize.

## Introduction and background

**Figure 1:**
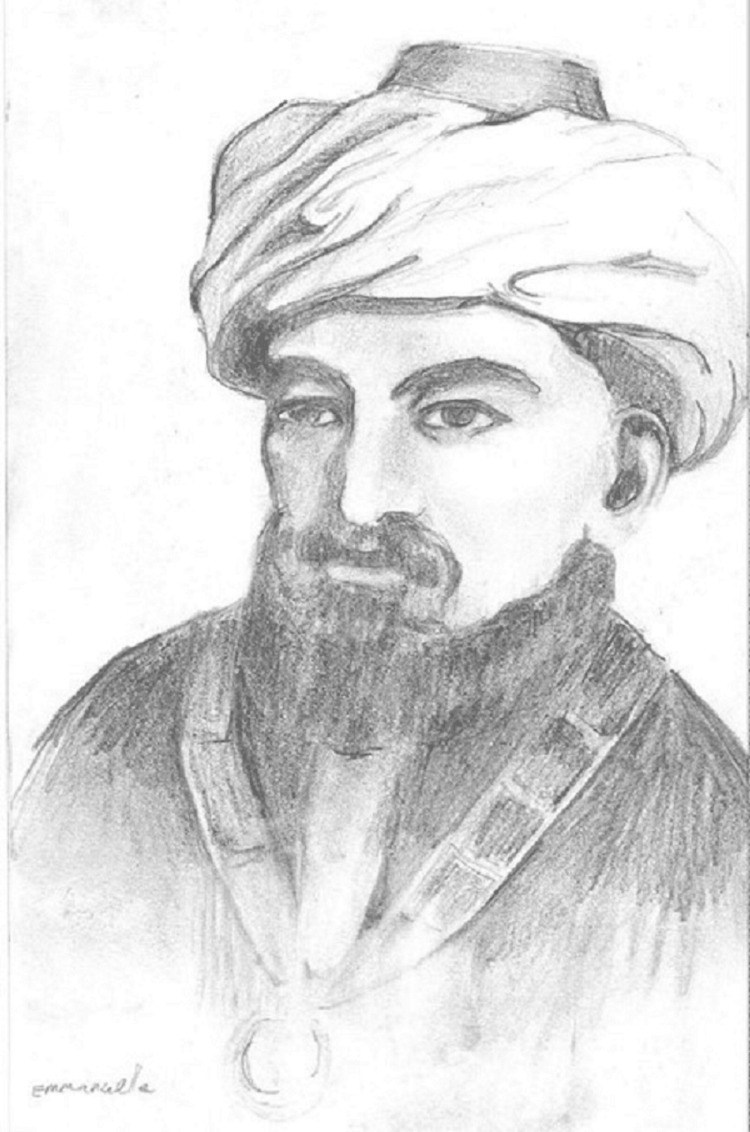
Rabbi Moshe Ben Maimon, Better Known as Rambam Drawing by Emmanuelle Herbalin with permission obtained from her.

The primary purpose of this article is to highlight the indisputable contribution of Rambam Rabbi Moshe Ben Maimon, known as "Maimonides" (the Hebrew name is Rambam) (Figure 1). He was born in Cordova, Spain, in 1138 and died in Fustat, today known as old Cairo, Egypt, in 1204. As a philosopher, adjudicator of Jewish law, physician, and scientist, Rambam played a significant role in many of these fields during the Middle Ages. His understanding of Jewish religious laws empowered him to explore diverse perspectives. The morals and ethics of Judaism are wide-ranging.

Moreover, Rambam possessed a tremendous understanding of the human body. Consequently, he was esteemed and respected not only by Jews but also by other religions and nationalities. There is a famous saying describing Rambam which is as follows: "From Moses (Moses, the prophet) to Moshe (Moses, the Rambam), there were none like Moses". He was nicknamed the "Big Eagle". Rambam was called "Mossa Ibn Maimon by the Arabs" and "Maimonides" by the Europeans, a derivative of the Greek word "Moiasis Maimonidis". Herein, we focus solely on Rambam's contributions to medicine since his work, activities, and knowledge are very complex to summarize [1-3]. Throughout his life, Rambam focused on several areas, despite their differences, and integrated them into his medical outlook, thus expressing his uniqueness.

## Review

Rambam’s life and career

Early Years

**Figure 2:**
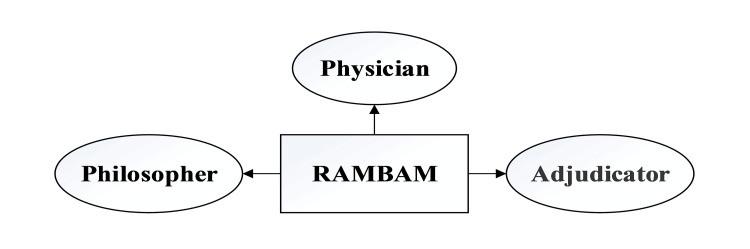
Rambam’s disciplines This image has been created by the authors using Microsoft Visio software (Microsoft Corp., Redmond, WA).

As mentioned, Rambam was born in Cordova (currently in Spain) in 1138. Spain had been part of the Almorvid empire for those years, at the end of the golden age of Jewish culture in Iberia (Cordova was under Muslim rule in those years) [4-5]. After he and his family were expelled from the country for refusing to convert to Islam, he lived in Morocco and Egypt. Rambam's father taught him from the Torah early on since he was a Jew. As a result of reading ancient Greek philosophy through Arabic translations and studying Islamic culture, he showed a deep interest in science [6]. Figure 2 summarizes the important areas of activity of Rambam's life.

Rambam, The Physician

Rambam trained in medicine in Spain and Fez, Morocco. He also learned Greek and Arabic medicine, practicing humourism principles in the tradition of Galen. Instead of blindly accepting authority, he relied on his own observations and experience. He describes some of the most known health conditions such as diabetes, hepatitis, asthma, and pneumonia [7].

The following are some of his most famous books in the field of medicine: *The Eight Chapters of Maimonides on Ethics*, which focuses on the body and soul of human beings [8]; *The Regimen of Health*, which relates to health and medicine, especially psychosomatic connections in human life, the physical state, and its relationship with emotions (known now as the body-mind connection) [9]; *The Medical Works of Moses Maimonides* (17 vols., 2002-2021), which contains teachings (1500) in all fields of medicine based on Galen’s theory [10]; *Commentary on Hippocrates' Aphorisms *which is on* *Hippocrates, who was was Rambam's favorite personality. This book deals with the concepts of health, medicine, and illness as well as Hippocrates' attitude toward these subjects relating to health, hygiene, and leadership [11]; *Galen's Letters*: Galen focused on diseases and their treatment. Rambam accepted his suppositions and believed the patient could be cured [12]; *Glossary of Drug Names*, which is an anthology of 2,500 drugs and their indications for use by Rambam. An impressive and rare collection, when it was published, dealing with medicine, the soul, and drugs. The book has been published in Arabic, Greek, Armenian, French, German, and Spanish [3].

As the private physician of King Saladin, who established the Ayyubid dynasty, Rambam demonstrated respect for his patients' autonomy and intercultural awareness while interacting with patients [13]. Table 1 summarizes Rambam’s most famous books in the field of medicine.

**Table 1:** Rambam’s most famous books in the field of medicine which are used by physicians

Book	Year of issue	Publisher	Reference number
The Medical Works of Moses Maimonides	2002	Brill	[3]
The Eight Chapters of Maimonides on Ethics: (Shemonah Perakim) (Vol. 7)	1912	Columbia University Press	[8]
Maimonides, Rabbi, Jurist, Philosopher, Astronomer, Community Leader. In Healers and Achievers: Physicians Who Excelled in Other Fields and the Times in Which They Lived	2012	Xlibris Corp	14

Rambam, The Adjudicator

After wandering the world, Rambam settled in Fustat (near Cairo, Egypt). The school he studied in was affiliated with a synagogue. Due to his contributions to the Jewish community in Egypt, Rambam became its head [1]. Rambam, according to his books, was also one of the most important linguists in the Jewish world. There are several important works that he wrote. These works are *Mishneh Torah *where Rambam composed a code of Jewish law that is comprehensive and deep. In this work, the Talmud's binding laws are gathered. It contains 14 books that make it easier for Jews of his time to understand the complex nature of Jewish rules and regulations they had adopted. Rabbi Yosef Karo wrote about him, “Who will dare force communities that follow the Rambam to follow any other decision (of the Jewish law), early or late? Rambam is the greatest of decisions, and all the communities of the land of Israel and the Arbistan and the Maghreb practice according to his word and accept him as their rabbi” [3].

Rambam, The Philosopher

In addition to this discipline, Rambam was a philosopher who contributed many works to the field. In addition to studying Arabic Muslim philosophers and interacting with Arabian teachers, he was influenced by Aristotle. Rambam's "13 Principles of Faith" described what he considered mandatory beliefs for Judaism: The existence of God; God’s unity and indivisibility into elements; God’s spirituality and incorporeality; God’s eternity; God alone should be the object of worship; Revelation through God’s prophets; The preeminence of Moses among the prophets; That the entire Torah (both written and oral law) is of divine origin and was dictated to Moses by God on Mount Sinai; The Torah, given by Moses, is permanent and will not be replaced or changed. God’s awareness of all human actions and thoughts; the Reward of righteousness and punishment of evil; The coming of the Jewish Messiah; and the resurrection of the dead [14-16]. Rambam has three major practice areas in his biography. Each one is complicated, but he found special ways to combine them.

**Figure 3:**
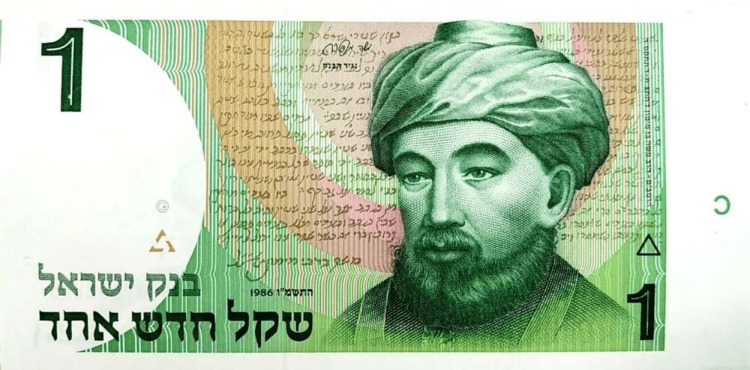
Rambam’s portrait on a currency bill of 1,000 shekels This photograph was taken by David Ezra from a private collection of Jacob Liderman with his permission.

Rambam is depicted on one of Israel's currency bills (Figure 3).

## Conclusions

The three monotheistic religions, Judaism, Islam, and Christianity, carry Rambam’s legacy worldwide. Today, Rambam is the name of many streets, especially in Israel. There is a large hospital in Haifa called Rambam, which is the largest in the north of Israel. There are many institutions in Israel, especially religious institutes. Rambam is considered a pioneer in medicine and philosophy, and a religious arbiter because of his activities. His contribution, especially to medicine, was unprecedented and important to the whole world.

## References

[1] Halbertal M (2013). Maimonides: Life and Thought.

[2] Mizrahi A (2011). The soul and the body in the philosophy of the Rambam. Rambam Maimonides Med J.

[3] Bos G (2021). Medical Aphorisms: Glossary & Indexes. The Medical Works of Moses Maimonides. Brill.

[4] Davidson HA (2005). Moses Maimonides: The Man and His Works.

[5] Stroumsa S (2014). On Maimonides and on Logic. Historical Studies in Science and Judaism.

[6] Kraemer JL (2010). Maimonides: The Life and World of One of Civilization's Greatest Minds. Rambam Maimonides Med J.

[7] Rosner F (2002). The life of Moses Maimonides, a prominent medieval physician. Einstein Quart J Biol Med.

[8] Maimonides M (1912). The Eight Chapters of Maimonides on Ethics: Shemonah Perakim Vol. 7.

[9] Segal I, Blazer S (2020). The Maimonides model for a regimen of health: a comparison with the contemporary scenario. Rambam Maimonides Med J.

[10] (1992). Stephanus (of Alexandria): Commentary on Hippocrates’ Aphorisms. Berlin, Akademie-Verlag.

[11] Kottek S (2009). Critical remarks on medical authorities: Maimonides’ commentary on Hippocrates’ aphorisms. Traditions of Maimonideanism. Traditions of Maimonideanism.

[12] Nutton V (1979). Galen on Prognosis.

[13] Yasin L, Stapleton GR, Sandlow LJ (2019). Medical professionalism across cultures: a literature review. MedEdPublish (2016).

[14] Bloch RS (2012). Maimonides, Rabbi, Jurist, Philosopher, Astronomer, Community Leader. Healers and Achievers: Physicians Who Excelled in Other Fields and the Times in Which They Lived.

[15] (2018). Moses Maimonides and His Time.

[16] Blumenthal DR (2014). Maimonides’ philosophic mysticism. David R. Blumenthal: Living with God and Humanity.

